# Profiles of Odd- and Branched-Chain Fatty Acids and Their Correlations With Rumen Fermentation Parameters, Microbial Protein Synthesis, and Bacterial Populations Based on Pure Carbohydrate Incubation *in vitro*

**DOI:** 10.3389/fnut.2021.733352

**Published:** 2021-09-22

**Authors:** Hangshu Xin, Nazir Ahmad Khan, Xin Liu, Xin Jiang, Fang Sun, Shuzhi Zhang, Yukun Sun, Yonggen Zhang, Xiang Li

**Affiliations:** ^1^College of Animal Science and Technology, Northeast Agricultural University, Harbin, China; ^2^Department of Animal Nutrition, The University of Agriculture Peshawar, Peshawar, Pakistan; ^3^Wellhope Feed Company Limited, Shenyang, China; ^4^Department of Ruminant Nutrition, Institute of Animal Husbandry of Heilongjiang Academy of Agricultural Sciences, Harbin, China

**Keywords:** odd- and branched-chain fatty acids, rumen fermentation products, microbial protein, volatile fatty acids, bacterial population

## Abstract

The objectives of this study were to evaluate changes in profiles of odd- and branched-chain fatty acids (OBCFA), including pentadecanoic acid (C15:0), 13-methyltetradecanoic acid (*iso*-C15:0), 12-methyltetradecanoic acid (*anteiso*-C15:0), 14-methylpentadecanoic acid (*iso*-C16:0), heptadecanoic acid (C17:0), 15-methylhexadecanoic acid (*iso*-C17:0), and 14-methylhexadecanoic acid (*anteiso*-C17:0) during *in vitro* fermentation of pure carbohydrates mixtures in the buffer-rumen fluid. The second objective was to correlate the changes in the OBCFA profile to the corresponding changes in ruminal fermentation parameters, microbial crude protein (MCP) synthesis, and bacterial populations. Five pure carbohydrates mixtures containing different cellulose: starch (C:S) ratios, i.e., 0:100, 25:75, 50:50, 75:25, and 100:0, were incubated for 6, 12, 18, and 24 h *in vitro*. The results showed that there was significant interaction (*P* < 0.05) between C:S and incubation time for changes in all OBCFA profiles, except iso-C17:0. The highest concentration of total OBCFA (3.94 mg/g dry matter; DM) was observed in the residues after 24 h of fermentation when the C:S was 0:100, while the lowest concentration of OBCFA (1.65 mg/g DM) was produced after 6 h of incubation when the C:S was 50:50. The correlation analysis revealed that the concentration of iso-C16:0 might be a potential marker for the estimation of total volatile fatty acids (ρ = 0.78) and MCP synthesis (ρ = 0.82) in the rumen. Compared to starch degrading bacteria, cellulolytic bacteria had stronger correlations with OBCFA concentrations, and the strongest correlation was found between the population of *Ruminococcus flavefaciens* with C15:0 concentration (ρ = 0.70). Notably, this is the first paper reporting relationship between OBCFA with rumen fermentation products and microbial protein synthesis based on fermentation of pure carbohydrates mixtures *in vitro*, and thus avoid confounding interference from dietary protein and fat presence in the *in vivo* studies. However, more in-depth experiments are needed to substantiate the current findings.

## Introduction

Odd- and branched-chain fatty acids contents are promising indices for predicting ruminal fermentation characteristics and microbial matter leaving the rumen (1–3). The contents of odd- and branched-chain fatty acids (OBCFA) include pentadecanoic acid (C15:0), 13-methyltetradecanoic acid (*iso*-C15:0), 12-methyltetradecanoic acid (*anteiso*-C15:0), 14-methylpentadecanoic acid (*iso*-C16:0), heptadecanoic acid (C17:0), 15-methylhexadecanoic acid (*iso*-C17:0), and 14-methylhexadecanoic acid (*anteiso*-C17:0). The OBCFA are present in microbial membrane lipids (4), and its profile reflects the microbial composition and as such, ruminal fermentation characteristics.

Theoretically, OBCFA is produced from 2-carbon elongation of propionate (C15:0 and C17:0), isobutyrate (*iso*-C14:0 and *iso*-C16:0), isovalerate (*iso*-C15:0 and *iso*-C17:0), and 2-methyl-butyrate (*anteiso*-C15:0 and *anteiso*-C17:0) with the incorporation of malonyl-CoA fatty acid synthetase, which provides the 2-carbons for elongation (4, 5). Therefore, it is not surprising that several studies have reported close relationships between OBCFA and ruminal volatile fatty acids (VFA) composition both *in vitro* (1) and *in vivo* (3). For example, the bacterial OBCFA explained 80% of the variations in acetate, propionate, and butyrate under an *in vitro* culture system (1). However, the study of French et al. (6) demonstrated that infusion of large amounts of VFA failed to alter OBCFA composition of ruminal content in mid-lactation dairy cows.

Earlier studies on the investigations of OBCFA profile have used a variety of natural feedstuffs, and the effect of the content and composition of dietary fat on OBCFA composition was always one of the main components of these studies (1, 6). The variation in content and composition of dietary fat might likely have altered the OBCFA profile by changing the microbial population in the rumen. As a result, these findings could not be attributed clearly to individual dietary constitutes such as starch and fiber; thus, may not extend well to different feedstuffs.

Since most OBCFA arise from microbial membrane lipids (4), greater concentrations of these fatty acids indicate greater microbial yield. A preliminary study (7) has confirmed that the ratios of purine bases:OBCFA (1.16) and uracil:OBCFA (0.34) were constant in mixed rumen bacteria of dairy cows. Similarly, our preliminary study confirmed that concentrations of ruminal OBCFA, especially those of odd-chain fatty acids and isomers of 15 carbon, were significantly correlated with pyrimidine, cytosine, and total nucleic acid bases in dairy cows (8). Although these nucleic acids are of microbial origin and widely used to quantify bacterial protein yield, they are not reliable indices alone because their ratio with nitrogen (N) content considerably varies between solid-associated bacteria and liquid-associated bacteria in the rumen (9). To the best of our knowledge, none of the previous studies has measured the true synthesis of microbial crude protein (MCP) in these experiments. Therefore, the relationship between OBCFA concentration and MCP yield has not been well-determined.

In addition, different bacteria consist of different OBCFA compositions in the rumen (10, 11). As well-reviewed by the study of Vlaeminck et al. (12), fibrolytic bacteria, e.g., *Ruminococcus flavefaciens* and *Ruminococcus albus*, are usually enriched in *iso*-fatty acids, while starch degrading microorganisms, e.g., *Selenomonas ruminantium, Ruminobacter amylophilus*, and *Streptococcus bovis*, contain a relatively greater amount of linear odd-chain fatty acids and a small amount of branched-chain fatty acids. In comparison, strains of *Butyrivibrio* have a more heterogeneous profile of OBCFA (11). Regarding the relationship of OBCFA concentrations with bacterial abundance in the rumen, little information is available, although data from our preliminary work indicated that C17:0 can be used to estimate the cellulolytic bacterial population in mixed rumen bacteria. However, more in-depth studies are required to build up a solid conclusion.

In this study, pure carbohydrates, i.e., cellulose and starch, were selected as fermentation substrates to avoid confounding effects of dietary fat on OBCFA synthesis during rumen fermentation. We hypothesized that the profiles of OBCFA would be different during ruminal fermentation of substrates with different ratios of cellulose and starch (C:S), and the variation in OBCFA composition might be correlated to changes in rumen fermentation characteristics and MCP synthesis *in vitro*. Therefore, the objectives of this study were to investigate changes in (1) OBCFA profiles, (2) rumen fermentation parameters, (3) MCP synthesis, and (4) relevant bacterial populations during *in vitro* rumen fermentation of substrates with different C:S ratios. The second objective was to correlate the changes in the OBCFA profile to the corresponding changes in ruminal fermentation parameters, MCP synthesis, and bacterial populations.

## Materials and Methods

The animal use protocol was approved by the Animal Care and Use Committee, Northeast Agricultural University (Harbin, China). All the procedures were conducted, following the University standard biosecurity rules.

### *In vitro* Incubation and Sample Collection

Carbohydrate mixtures containing different ratios of pure cellulose and starch (C:S = 0:100, 25:75, 50:50, 75:25, and 100:0) were obtained from Macklin (Macklin Biochemical Co., Ltd, Shanghai, China), and used as fermentation substrates for *in vitro* incubations. From each of the five substrates, subsamples (1 g) were weighed and transferred into 150 ml culture flasks for subsequent *in vitro* incubation for 6, 12, 18, and 24 h in a buffered-rumen fluid. For each incubation period, three replicate samples of each substrate were used. The incubations were carried out in two replicate runs within 2 weeks.

Rumen fluid was obtained from three rumen-fistulated Holstein cows in warm (39°C) thermostat bottles which were pre-flushed with CO_2_. The cows were fed a balanced ration (Table 1), formulated according to the NRC (13), at 08:00 and 16:00 h daily. The rumen fluid was strained through four layers of cheesecloth and the residues were discarded. The filtered fluid was transferred to a 4 L glass jar and mixed with pre-warmed (39°C) buffer solution in 1:2 (V/V) under a continuous supply of CO_2_ to ensure an anaerobic environment. The buffer solution was prepared according to the study of Wang et al. (14) and contained NH_4_HCO_3_, NaHCO_3_, Na_2_HPO_4_, KH_2_PO_4_, MgSO_4_•7H_2_O, CaCl_2_•2H_2_O, MnCl_2_•4H_2_O, CoCl_2_•6H_2_O, FeCl_3_•6H_2_O, C_12_H_6_NO_4_Na, trypticase peptone, C_3_H_7_NO_2_S•HCl•H_2_O, and Na_2_S•9H_2_O. The buffered rumen fluid was transferred to the dispenser with a volume range of 100 ml and connected with CO_2_. Before adding the buffered rumen fluid, the incubation flasks containing the samples were flushed with CO_2_. Afterward, 100 ml of the buffered-rumen fluid was added through the walls to each flask. The flasks were slowly and smoothly stirred to prevent adhesion of particles above the fluid to the bottle walls. The flasks were incubated in a shaking water bath at 39°C with 40–50 movements per minute for 24 h. Triplicate flasks containing only mixed buffered-rumen fluid were also incubated in each run as blank control. After 6, 12, 18, and 24 h of incubation, a total of 18 flasks (15 treatments and 3 blanks) were taken out of the incubator and immediately placed in the ice to end the fermentation. Subsequently, approximately 5 ml of fermented inoculum from each flask was subsampled for the measurements of pH value (Sartorius, pH-Meter PB-10, Goettingen, Germany), ammonia nitrogen (NH_3_-N), and volatile fatty acids (VFA) concentrations. Then another 4 ml of inoculum was snap-frozen in LN and stored at −80°C for further analysis of rumen bacterial populations. Finally, the remaining fermented samples were freeze-dried, weighed, and analyzed for OBCFA and MCP concentrations.

**Table 1:** Ingredients and chemical compositions of diets for donor animals.

**Ingredients (%)**		**Chemical composition**	
Chinese wildrye	43.0	${\text{NE}}_{\text{L}}^{\text{b}}$, MJ/kg dry matter (DM)	5.40
Corn silage	16.0	Crude protein, %DM	14.4
Corn	13.0	Neural detergent fiber, %DM	49.2
Wheat bran	4.0	Acid detergent fiber, %DM	30.6
Molasses	1.0	Ca, %DM	0.60
Soybean meal	3.0	P, %DM	0.40
Dried distillers grain	5.0		
Cottonseed meal	2.0		
Corn gluten feed	7.5		
Corn germ meal	5.0		
Premix^a^	0.5		
Total	100.0		

a *Contained the following per kg of the premix: V_A_ 8,000,000 IU, V_D_ 700,000 IU, V_E_ 10,000 IU, Fe 1,600 mg, Cu 1,500 mg, Zn 10,000 mg, Mn 3,500 mg, Se 80 mg, I 120 mg, Co 50 mg.*

*^b^ NE_L_ is a calculated value and other nutrient levels are measured values*.

### Measurement of Odd- and Branched-Chain Fatty Acids

The OBCFA were determined according to the procedure described by the study of Sukhija and Palmquist (15). In brief, around 200 mg of freeze-dried fermentation mixture was weighed and added into each nylon-cap glass tube containing 2 mg of non-adecanoic acid (C19:0, internal standard; Macklin Biochemical Co., Ltd, Shanghai, China). Then the samples were treated with 3 ml of 5% methanolic HCl and incubated at 70°C for 2 h. After cooling to room temperature, 5 ml of 6% K_2_CO_3_ was added which was followed by 2 ml of benzene and vortexed for 30 s. Following centrifugation at 1,500 rpm for 5 min, the upper organic phase was transferred, dried with N_2_, and reconstituted in 1 ml of benzene for gas chromatography (GC) analysis.

The methylated fatty acids were analyzed by Shimadzu GC analyzer (GC-2010, Shimadzu, Tokyo, Japan), equipped with flame ionization detector (FID), an Equity™-1 capillary column (15 m ×0.1 mm ×0.1 μm; Supelco, Inc., Sigma-Aldrich, 28039-U), and N_2_ as the carrier gas. The injector and detector were maintained at 280°C. Individual OBCFA was identified according to the peak and retention time of fatty acid standards, including *iso*-C14:0, *iso*-C15:0*, anteiso*-C15:0, C15:0, *iso*-C16:0, *iso*-C17:0, *anteiso*-C17:0, C17:0, and *iso*-C18:0 (Larodan Fine Chemicals, Malmo, Sweden).

### Analyses of Ruminal Fermentation Parameters

The concentration of NH_3_-N was determined using the phenol-hypochlorite colorimetric method according to the study of Broderick and Kang (16). The VFA concentrations were measured by using GC-2010 (Shimadzu Laboratory Supplies Co., Ltd, Shanghai, China) equipped with a flame-ionization detector and a capillary column (HP-Innowax, 19091N-133, Agilent Technologies, Santa Clara, CA), as described by the study of Zhang et al. (3).

The MCP synthesis was estimated according to the study of Hall and Herejk (17). Briefly, 300 mg of freeze-dried fermentation residue was weighed, transferred into a 50 ml flask containing 10 ml of trichloroacetic acid (TCA; 19.4%), and the mixtures were then placed on ice for 45 min. Following centrifugation at 7,719 × g for 20 min, the whole flask content was filtered through Whatman 541 filter paper (Fisher Scientific, Atlanta, GA). Subsequently, the filtrate was filtered again through a Whatman GF/A glass fiber filter (Fisher Scientific, Atlanta, GA) using 2% TCA to rinse the flask. Both Whatman 541 and GF/A filters were dried at 55°C overnight and then subjected to Kjeldahl analysis.

### DNA Extraction and qPCR Analysis

Total DNA of ruminal microbes was extracted using the cetyl trimethyl ammonium bromide (CTAB) plus bead-beating method (18). The air-dried DNA pellet was re-suspended in Tris-EDTA buffer (10 mM Tris-HCl, 1 mM EDTA, pH 8) and was stored at −20°C until further processing (3). The DNA purity (A260/280) was detected using a microplate spectrophotometer (SpectraMax 190, Sunnyvale, USA). Then the DNA samples were diluted to 10 ng/μl for real-time qPCR amplification. The qPCR primers used in this study (Table 2) were assembled according to previous studies (19–21) and obtained from Sangon Biotech Co. Ltd. (Shanghai, China). By following the manual instruction, the qPCR was performed using Takara SYBR® Premix Ex Taq. Synthesis Kit (Code No. RR420A, Takara, Dalian, China). The relative abundance of bacterial species was expressed as a proportion in total estimated bacterial 16S rDNA by the calculation of relative abundance = 2^−(*Cttarget*−*Cttotalbacteria*)^, where Ct represents threshold cycle (23).

**Table 2:** Primers used for real-time PCR quantification.

	**Primer sequence (5^**′**^-3^**′**^)**	**Product size (bp)**	**References**
General bacteria	F:CGGCAACGAGCGCAACCC R:CCATTGTAGCACGTGTGTAGCC	130	(19)
*Ruminococcus albus*	F:GTTTTAGGATTGTAAACCTCTGTCTT R:CCTAATATCTACGCATTTCACCGC	273	(20)
*Ruminococcus flavefaciens*	F:GATGCCGCGTGGAGGAAGAAG R:CATTTCACCGCTACACCAGGAA	278	(20)
*Butyrivibrio fibrisolvens*	F:TAACATGAGTTTGATCCTGGCTC R:CGTTACTCACCCGTCCGC	113	(20)
*Selenomonas ruminantium*	F:CAATAAGCATTCCGCCTGGG R:TTCACTCAATGTCAAGCCCTGG	138	(21)
*Streptococcus bovis*	F:TTCCTAGAGATAGGAAGTTTCTTCGG R:ATGATGGCAACTAACAATAGGGGT	127	(21)
*Ruminobacter amylophilus*	F:CTGGGGAGCTGCCTGAAT R: CATCTGAATGCGACTGGTTG	100	(22)

### Statistical Analysis

The data were analyzed using PROC MIXED model in SAS (version 9.4, SAS Institute Inc., Cary, NC, USA). The model included the fixed effects of different carbohydrate substrates (C:S ratio = 0:100, 25:75, 50:50, 75:25, and 100:0), incubation time (6, 12, 18, and 24 h), their interaction (C:S ratio × incubation time), and random effect of experimental run. Statistical significance was declared at *P* < 0.05. To investigate the relationships between OBCFA and rumen parameters, a Spearman's rank correlation analysis was performed in this study. A significant correlation was defined *P* < 0.05 and a strong correlation was defined when −0.5 > ρ > 0.5 and *P* < 0.05, where ρ is defined as the Spearman rank-order correlation coefficient. In this study, only significant correlations are shown.

## Results

### Effect of Different Ratios of Cellulose and Starch on Ruminal OBCFA Synthesis During 24 h of Incubation *in vitro*

Data on the effect of the different pure C:S ratios on ruminal OBCFA synthesis (mg/g DM) during 24 h incubation *in vitro* are summarized in Table 3. All measured OBCFA including *iso*-C15:0, *anteiso*-C15:0, C15:0, *iso*-C16:0, *iso*-C17:0, and *anteiso*-C17:0 was altered (*P* < 0.001) by C:S ratios and incubation time. However, the significant interactions (*P* < 0.01) between C:S ratios and incubation time (except for *iso*-C17:0), prohibit unequivocal comparison of main effects. Among OBCFA, *anteiso*-C15:0 and C15:0 were the top two abundant fatty acids, accounting for 17.6–23.6% and 15.4–38.1% of the total OBCFA, respectively. The total OBCFA concentration ranged from 1.65 to 3.94 mg/g DM in different residual samples after *in vitro* incubations. The highest concentration of total OBCFA (3.94 mg/g DM) was recorded in the fermented mixture after 24 h fermentation when the C: S ratio was 0:100, while the lowest concentration of OBCFA (1.65 mg/g DM) was produced after 6 h of incubation when the C:S ratio was 50:50. The concentrations of C15:0, *iso*-C16:0, *iso*-C17:0, *anteiso*-C17:0, and C17:0 were linearly and/or quadratically changed (*P* < 0.05) as cellulose proportion increased to 100% in the substrates. The OBCFA concentration varied quadratically (*P* < 0.05), wherein it decreased as the cellulose ratio increased up to 50%, and then increased gradually.

**Table 3:** Effect of different ratios of cellulose and starch (C:S) on ruminal OBCFA synthesis (mg/g DM) during 24 h of incubation *in vitro*.

	**Time (h)**	***iso*-C15:0**	***anteiso*-C15:0**	**C15:0**	***iso*-C16:0**	***iso*-C17:0**	***anteiso*-C17:0**	**C17:0**	**TC15^a^**	**TC17^b^**	**TIFA^c^**	**TAFA^d^**	**TOCFA^e^**	**TBCFA^f^**	**OBCFA^g^**
C:S = 0:100^h^	6	0.22	0.54	0.56	0.31	0.31	0.37	0.35	1.31	1.02	0.84	0.90	0.90	1.74	2.64
	12	0.23	0.58	0.51	0.33	0.36	0.38	0.27	1.29	1.00	0.91	0.96	0.78	1.87	2.65
	18	0.24	0.60	0.55	0.38	0.39	0.46	0.31	1.39	1.17	1.01	1.06	0.87	2.07	2.93
	24	0.31	0.76	0.69	0.48	0.47	0.80	0.43	1.75	1.71	1.26	1.56	1.12	2.82	3.94
C:S = 25:75	6	0.19	0.43	0.33	0.25	0.32	0.30	0.32	0.95	0.94	0.76	0.73	0.65	1.49	2.14
	12	0.21	0.56	0.42	0.26	0.33	0.35	0.25	1.19	0.92	0.80	0.90	0.66	1.71	2.37
	18	0.24	0.64	0.62	0.34	0.41	0.49	0.33	1.50	1.24	0.99	1.13	0.95	2.13	3.07
	24	0.30	0.75	0.59	0.42	0.45	0.64	0.40	1.64	1.48	1.17	1.39	0.99	2.56	3.55
C:S = 50:50	6	0.14	0.29	0.34	0.17	0.26	0.24	0.22	0.76	0.72	0.57	0.53	0.56	1.09	1.65
	12	0.21	0.57	0.54	0.26	0.35	0.44	0.24	1.32	1.03	0.82	1.01	0.79	1.83	2.62
	18	0.25	0.59	0.61	0.23	0.35	0.40	0.23	1.45	0.98	0.83	0.98	0.85	1.81	2.66
	24	0.33	0.76	0.61	0.38	0.40	0.83	0.29	1.70	1.52	1.11	1.58	0.91	2.69	3.60
C:S = 75:25	6	0.18	0.40	0.32	0.18	0.32	0.25	0.23	0.91	0.81	0.68	0.66	0.55	1.34	1.89
	12	0.28	0.71	1.03	0.30	0.35	0.34	0.27	2.01	0.95	0.92	1.04	1.30	1.96	3.26
	18	0.37	0.76	1.10	0.25	0.41	0.31	0.31	2.24	1.04	1.03	1.08	1.42	2.11	3.52
	24	0.37	0.82	1.04	0.33	0.44	0.35	0.44	2.24	1.24	1.15	1.18	1.48	2.32	3.81
C:S = 100:0	6	0.14	0.31	0.37	0.11	0.32	0.26	0.24	0.82	0.81	0.57	0.57	0.61	1.13	1.74
	12	0.23	0.63	0.94	0.18	0.33	0.28	0.29	1.80	0.90	0.74	0.91	1.23	1.64	2.87
	18	0.29	0.65	1.15	0.15	0.35	0.25	0.30	2.09	0.91	0.79	0.91	1.45	1.70	3.15
	24	0.29	0.64	1.25	0.16	0.38	0.26	0.32	2.18	0.95	0.83	0.90	1.56	1.72	3.28
SEM		0.015	0.034	0.049	0.016	0.018	0.025	0.019	0.080	0.037	0.038	0.045	0.057	0.073	0.110
	C:S	<0.0001	<0.0001	<0.00011	<0.0001	0.0011	<0.0001	<0.0001	<0.0001	<0.0001	<0.0001	<0.0001	<0.0001	<0.0001	<0.0001
*P*	Time	<0.0001	<0.0001	<0.0001	<0.0001	<0.0001	<0.0001	<0.0001	<0.0001	<0.0001	<0.0001	<0.0001	<0.0001	<0.0001	<0.0001
	C:S × Time	0.0003	0.001	<0.0001	<0.0001	0.13	<0.0001	0.0006	<0.0001	<0.0001	0.01	<0.0001	<0.0001	<0.0001	<0.0001
Polynomial contrast-C:S	Linear	0.11	0.44	<0.0001	<0.0001	0.02	<0.0001	0.0002	<0.0001	<0.0001	<0.0001	<0.0001	<0.0001	<0.0001	0.22
	Quadratic	0.37	0.78	<0.0001	0.03	0.54	0.0002	0.001	0.001	0.54	0.32	0.02	<0.0001	0.05	0.04
Polynomial contrast-Time	Linear	<0.0001	<0.0001	<0.0001	<0.0001	<0.0001	<0.0001	<0.0001	<0.0001	<0.0001	<0.0001	<0.0001	<0.0001	<0.0001	<0.0001
	Quadratic	0.27	0.0004	<0.0001	0.09	0.75	<0.0001	<0.0001	<0.0001	<0.0001	0.67	0.95	0.0005	0.79	0.08

a *TC15 = iso-C15:0 + anteiso-C15:0 + C15:0;*

b *TC17 = iso-C17:0 + anteiso-C17:0 + C17:0;*

c *TIFA = iso-C15:0 + iso-C16:0 + iso-C17:0;*

d *TAFA = anteiso-C15:0 + anteiso-C17:0;*

e *TOCFA = C15:0 + C17:0;*

f *TBCFA = iso-C15:0 + iso-C16:0 + iso-C17:0 + anteiso-C15:0 + anteiso-C17:0;*

g *OBCFA = iso-C15:0 + anteiso-C15:0 + C15:0 + iso-C16:0 + iso-C17:0 + anteiso-C17:0 + C17:0;*

h *C:S = cellulose:starch*.

The concentrations of *iso*-C15:0, *iso*-C16:0, *iso*-C17:0, total *iso*-fatty acids (TIFA), total *anteiso*-fatty acids (TAFA), total branched-chain fatty acids (TBCFA), and OBCFA linearly increased (*P* < 0.05), while the remaining OBCFA parameters were altered in both linear and quadratic fashions (*P* < 0.05) in all fermentation substrates, as incubation time increased within 24 h ruminal incubation *in vitro*.

### Effect of Substrates With Different Ratios of Cellulose and Starch on Ruminal Fermentation Parameters and Microbial Protein Synthesis During 24 h Incubation *in vitro*

Except for acetate, total VFA (TVFA), pH and MCP synthesis, and all measured ruminal fermentation parameters had significant interactions (*P* < 0.05) between C:S ratio and incubation time, prohibiting unequivocal comparison of main effects (Table 4). The molar proportions of propionate, isobutyrate, butyrate, isovalerate, and acetate/propionate altered in linear and quadratic fashions (*P* < 0.05), whereas TVFA concentration remarkably decreased linearly (*P* < 0.05) as the inclusion of cellulose in the substrates increased. The *in vitro* ruminal NH_3_-N concentrations increased (linear or quadratic; *P* < 0.05) when the starch supply reduced in the substrates. The microbial crude protein synthesis decreased linearly (*P* < 0.05) as C:S ratio increased. The total VFA concentration increased while pH value and NH_3_-N concentrations decreased linearly as incubation time increased (*P* < 0.05).

**Table 4:** Effect of different ratios of cellulose and starch (C:S) on ruminal fermentation parameters and MCP^a^ concentration during 24 h of incubation *in vitro*.

	**Time (h)**	**Acetate (mmol/mol)**	**Propionate (mmol/mol)**	**Isobutyrate (mmol/mol)**	**Butyrate (mmol/mol)**	**Isovalerate (mmol/mol)**	**TVFA (mmol/L)**	**Acetate/propionate**	**NH_**3**_-N (mg/dL)**	**pH**	**MCP (mg/g DM)**
C:S = 0:100^b^	6	553.9	220.1	8.78	206.4	10.9	55.5	2.52	1.38	6.50	119.9
	12	510.0	252.0	7.95	211.5	10.9	73.2	2.02	0.45	6.41	120.0
	18	510.6	231.2	8.82	229.8	12.4	88.1	2.22	0.31	6.33	133.8
	24	496.1	241.1	9.21	232.5	13.3	85.8	2.06	2.56	6.26	147.4
C:S = 25:75	6	546.6	222.7	9.24	209.4	12.0	53.1	2.45	4.94	6.64	93.2
	12	510.9	254.6	8.70	214.4	11.3	59.3	2.01	4.00	6.58	94.8
	18	507.4	258.2	8.44	206.9	11.5	78.0	1.97	0.69	6.48	99.4
	24	495.5	251.4	7.95	226.2	11.4	78.8	1.97	2.73	6.39	119.6
C:S = 50:50	6	547.2	221.4	9.30	209.6	12.5	52.9	2.48	7.83	6.71	97.2
	12	521.3	242.5	8.68	215.4	12.2	59.3	2.15	3.12	6.62	104.8
	18	521.2	241.9	7.88	209.8	10.7	68.4	2.16	4.20	6.55	108.0
	24	506.8	240.0	8.58	224.6	11.9	72.8	2.11	4.03	6.49	111.9
C:S = 75:25	6	533.0	220.8	10.61	221.1	14.5	44.1	2.41	11.19	6.78	76.2
	12	510.7	245.0	9.51	222.3	12.5	53.0	2.09	6.33	6.70	99.9
	18	504.9	260.6	8.48	215.5	10.4	68.5	1.94	3.35	6.55	107.9
	24	485.8	271.8	8.96	222.0	11.4	69.1	1.79	2.07	6.48	108.4
C:S = 100:0	6	536.1	240.4	13.31	192.2	18.0	35.1	2.23	17.05	6.85	69.5
	12	522.1	273.5	11.44	177.5	15.5	45.2	1.91	10.62	6.80	76.9
	18	498.5	318.2	9.78	160.5	13.1	54.9	1.57	7.06	6.67	86.2
	24	493.4	323.8	11.62	157.3	13.9	56.8	1.53	7.83	6.61	85.7
SEM		6.70	5.43	0.429	6.05	0.446	3.243	0.061	0.737	0.040	5.065
	C:S	0.03	<0.0001	<0.0001	<0.0001	<0.0001	<0.0001	<0.0001	<0.0001	<0.0001	<0.0001
*P*	Time	<0.0001	<0.0001	<0.0001	0.24	<0.0001	<0.0001	<0.0001	<0.0001	<0.0001	<0.0001
	C:S × Time	0.68	<0.0001	0.02	0.001	<0.0001	0.56	0.007	<0.0001	0.99	0.20
Polynomial contrast-C:S	Linear	0.12	<0.0001	<0.0001	<0.0001	<0.0001	<0.0001	<0.0001	<0.0001	<0.0001	<0.0001
	Quadratic	0.36	<0.0001	<0.0001	<0.0001	<0.0001	0.05	<0.0001	0.001	0.12	0.35
Polynomial contrast-Time	Linear	<0.0001	<0.0001	0.0002	0.38	<0.0001	<0.0001	<0.0001	<0.0001	<0.0001	<0.0001
	Quadratic	0.01	<0.0001	0.0002	0.17	<0.0001	0.004	<0.0001	<0.0001	0.88	0.90

a *MCP, microbial crude protein synthesis;*

b *C:S, cellulose:starch*.

### Effect of Substrates With Different Ratios of Cellulose and Starch on Bacterial Population Composition During 24 h Incubation *in vitro*

In the present study, three cellulolytic bacteria (*R. albus, R. flavefaciens*, and *B. fibrisolvens*) and three amylolytic bacteria (*S. ruminantium, R. amylophilus*, and *S. bovis*) populations were measured using the real-time qPCR system (Table 5). Among the bacterial population, *R. flavefacien* (2.23–29.28%) and *R. amylophilus* (0.3–28.91%) were the top two abundant bacteria during 24 h of *in vitro* incubation. The third predominant bacterium that is *S. ruminantium* which accounted for.42–1.11%, was not affected (*P* > 0.05) by C:S ratio and incubation time. Except for *S. ruminantium*, significant interactions (*P* < 0.05) between C:S ratio and incubation time was observed for all bacterial population. The proportions of investigated cellulolytic bacteria in the present *in vitro* study increased linearly or quadratically *(P* < 0.05), while those of *Ramylophilus* and *S. bovis* decreased (*P* < 0.05) as cellulose inclusion increased in the substrates. For each kind of mixed carbohydrate substrates, the relative abundances of all the bacteria (except *S. ruminantium*) had marked reductions overtime during the 24-h incubation, in linear or quadratic fashions (*P* < 0.05).

**Table 5:** Effect of different ratios of cellulose and starch (C:S) on ruminal bacteria populations^a^ during 24 h of incubation *in vitro*.

	**Time (h)**	***R. albus* (10^**–2**^%)**	***R. flavefaciens* (10^**–2**^%)**	***B. fibrisolvens (*%)**	***S. ruminantium* (%)**	***R. amylophilus* (%)**	***S. bovis* (10^**–2**^%)**
C:S = 0:100^b^	6	0.19	3.44	11.38	0.70	14.94	0.19
	12	0.10	0.20	14.78	0.67	14.82	0.09
	18	0.06	0.14	6.76	0.45	24.04	0.25
	24	0.09	0.42	2.23	0.61	9.83	0.25
C:S = 25:75	6	0.40	1.15	17.40	0.97	10.93	0.07
	12	0.03	0.32	4.14	0.52	16.72	0.17
	18	0.07	0.28	5.71	0.42	23.19	0.15
	24	0.07	0.71	3.89	0.45	8.34	0.68
C:S = 50:50	6	0.16	1.85	10.33	0.44	17.58	0.08
	12	0.23	1.58	6.77	0.80	28.91	0.20
	18	0.13	1.44	9.50	0.47	17.01	0.15
	24	0.09	2.12	5.84	0.58	13.00	0.29
C:S = 75:25	6	0.30	1.30	24.48	0.88	11.50	0.19
	12	0.27	3.56	9.74	0.90	17.02	0.47
	18	0.34	13.41	15.36	0.66	4.18	0.45
	24	0.56	7.64	8.48	0.86	1.82	0.40
C:S = 100:0	6	0.17	0.81	29.28	0.48	0.74	0.12
	12	0.22	1.70	9.47	0.70	0.79	0.14
	18	0.70	7.17	10.79	0.96	0.30	0.07
	24	1.48	21.76	8.50	1.11	0.53	0.04
SEM		0.001	0.020	0.023	0.002	0.032	0.001
	C:S	<0.0001	<0.0001	<0.0001	0.10	<0.0001	0.0001
*P*	Time	0.001	<0.0001	<0.0001	0.614	0.001	0.0004
	C:S × Time	<0.0001	<0.0001	0.001	0.18	0.02	0.0002
Polynomial contrast-C:S	Linear	<0.0001	<0.0001	<0.0001	0.03	<0.0001	0.25
	Quadratic	0.002	0.004	0.07	0.40	<0.0001	0.004
Polynomial contrast-Time	Linear	0.002	<0.0001	<0.0001	0.92	0.02	<0.0001
	Quadratic	0.01	0.05	0.01	0.49	0.0003	0.29

a *Bacteria population was measured as a proportion of the total estimated rumen bacterial 16S rRNA gene [relative quantification = 2^−(CT−targetbacteria−CT−totalbacteria)^];*

b *C:S, cellulose:starch*.

### Correlation of OBCFA Synthesis With Fermentation Parameters and Bacterial Populations During 24 h Incubation *in vitro*

**Figure 1:**
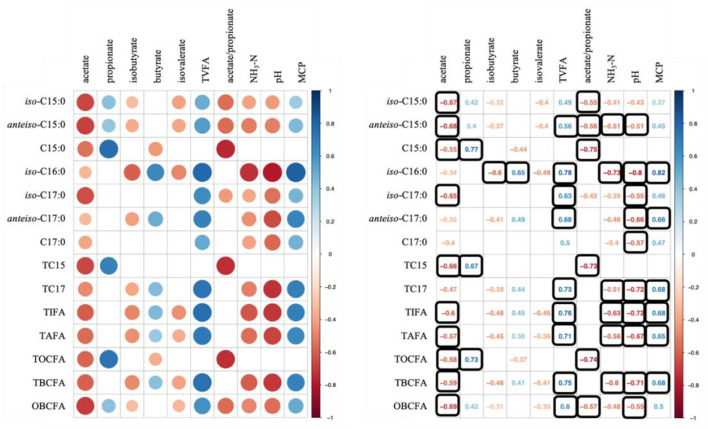
Correlation analysis between ruminal fermentation parameters/MCP and OBCFA concentrations during 24 h of incubation *in vitro*. Color in round shape represents significant correlations (*P* < 0.05) and black square means −0.50 > ρ > 0.50.

Data used for correlation analysis are summarized in Table 6. Figure 1 shows results of Spearman's rank correlation analysis between ruminal fermentation products and OBCFA synthesis during 24 h *in vitro* rumen fermentation of the different mixtures of pure carbohydrates. In general, strong correlations were observed between the molar proportion of acetate and concentration of OBCFA than between other individual VFA molar proportion and concentration of OBCFA (Figure 1). The acetate molar proportion was negatively correlated (*P* < 0.05; ρ = −0.32 to −0.69) with all measured OBCFA concentrations. Within these correlations, 10 were relatively stronger (ρ < −0.50). The molar proportion of propionate was positively correlated with concentrations of C15:0 (*P* < 0.05; ρ = 0.77), TC15 (TC15 = *iso*-C15:0 + *anteiso*-C15:0 + C15:0; *P* < 0.05; ρ = 0.67) and TOCFA (TOCF = C15:0 + C17:0; *P* < 0.05; ρ = 0.73), while the molar proportion of isobutyrate (*P* < 0.05; ρ = −0.60) and butyrate (*P* < 0.05; ρ = 0.65) had significant correlation with *iso*-C16:0 concentration. The concentration of TVFA had positive correlation with all measured OBCFA parameters (*P* < 0.05; ρ = 0.49–0.78), except C15:0, TC15 and TOCFA (*P* > 0.05).

**Table 6:** Summary statistics of variables utilized for the correlation analysis.

	**Mean**	**Standard deviation**	**Minimum**	**Maximum**
**OBCFA profile, mg/g DM**				
*iso*-C15:0	0.40	0.12	0.16	0.70
*anteiso*-C15:0	0.94	0.25	0.39	1.44
C15:0	1.09	0.56	0.46	2.48
*iso*-C16:0	0.42	0.12	0.18	0.64
*iso*-C17:0	0.58	0.10	0.33	0.79
*anteiso*-C17:0	0.62	0.22	0.34	1.46
C17:0	0.48	0.11	0.29	0.84
TC15^a^	2.43	0.88	1.05	4.25
TC17^b^	1.67	0.32	0.99	2.66
TIFA^c^	1.39	0.27	0.75	2.07
TAFA^d^	1.56	0.39	0.73	2.77
TOCFA^e^	1.57	0.62	0.79	3.09
TBCFA^f^	2.95	0.63	1.48	4.64
OBCFA^g^	4.52	1.10	2.30	7.10
**Ruminal fermentation parameters and MCP synthesis**				
Acetate, mmol/mol	515.6	21.3	482.5	572.3
Propionate, mmol/mol	251.6	29.0	212.2	340.1
Isobutyrate, mmol/mol	9.36	1.50	6.83	13.8
Butyrate, mmol/mol	208.2	22.4	148.6	244.2
Isovalerate, mmol/mol	12.5	1.91	9.44	18.4
TVFA, mmol/L	62.6	14.8	33.7	89.6
Acetate/propionate	2.08	0.28	1.43	2.59
NH_3_-N, mg/dL	5.09	4.29	0.30	18.5
pH	6.57	0.16	6.19	6.90
MCP, g/kg DM	160.7	22.1	110.1	205.7
**Ruminal bacterial population**				
*R. albus*, 10^−2^%	0.29	0.36	0.02	2.17
*R. flavefaciens*, 10^−2^%	3.60	5.63	0.10	28.40
*B. fibrisolvens*, %	10.89	7.37	1.78	31.86
*S. ruminantium*, %	0.68	0.32	0.25	1.51
*R. amylophilus*, %	11.84	9.57	0.27	44.44
*S. bovis*, 10^−2^%	0.23	0.21	0.04	1.10

a *TC15 = iso-C15:0 + anteiso-C15:0 + C15:0;*

b *TC17 = iso-C17:0 + anteiso-C17:0 + C17:0;*

c *TIFA = iso-C15:0 + iso-C16:0 + iso-C17:0;*

d *TAFA = anteiso-C15:0 + anteiso-C17:0;*

e *TOCFA = C15:0 + C17:0;*

f *TBCFA = iso-C15:0 + iso-C16:0 + iso-C17:0 + anteiso-C15:0 + anteiso-C17:0;*

g *OBCFA = iso-C15:0 + anteiso-C15:0 + C15:0 + iso-C16:0+ iso-C17:0 + anteiso-C17:0 + C17:0*.

Except C15:0, the concentrations of OBCFA were negatively correlated with NH_3_-N (*P* < 0.05; ρ = −0.39 to −0.73) and positively correlated with MCP synthesis (*P* < 0.05; ρ = 0.37–0.82). In our study, the strong correlation of MCP concentration was observed with iso-C16:0 (ρ = 0.82) and *anteiso*-C17:0 (ρ = 0.66) concentrations.

**Figure 2:**
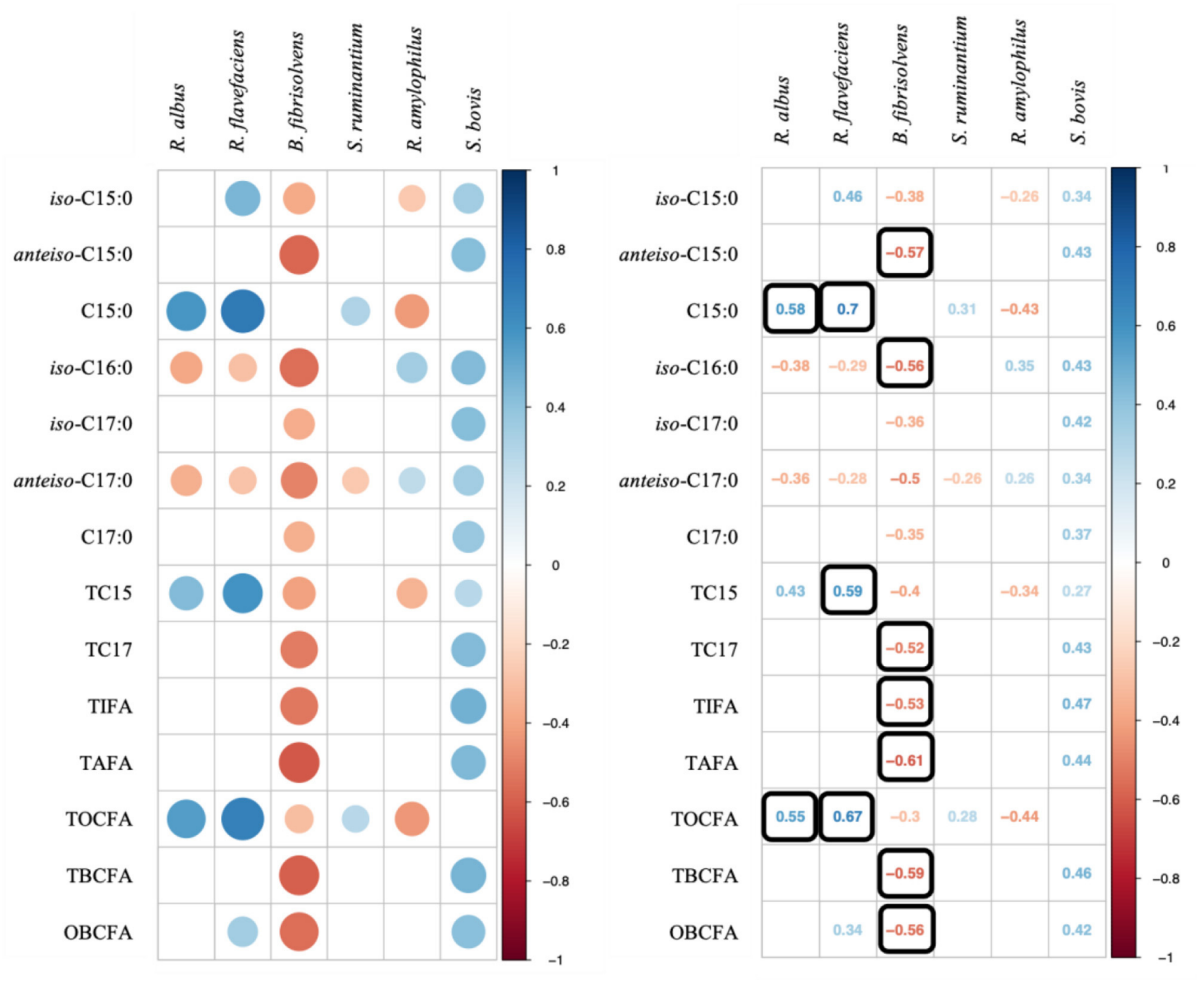
Correlation analysis on ruminal bacterial populations and OBCFA concentrations during 24 h of incubation *in vitro*. Color in Q22 round shape represents significant correlations (*P* < 0.05) and black square means −0.50 > ρ > 0.50.

We further investigated the correlations between OBCFA and bacterial populations (Figure 2). Stronger correlations were observed between OBCFA production and cellulolytic bacteria (*R. albus, R. flavefaciens*, and *B. fibrisolvens*) than between OBCFA and starch-degrading bacteria (*S. ruminantium, R. amylophilus*, and *S. bovis*). Notably, the relative abundance of *R. albus* (*P* < 0.05; ρ = 0.58) and *R. flavefaciens* (*P* < 0.05; ρ = 0.70) were positively correlated with C15:0, while that of *B. fibrisolvens* abundance was negatively correlated with *anteiso*-C15:0 (*P* < 0.05; ρ = −0.57) and *iso*-C16:0 (*P* < 0.05; ρ = −0.56). Although statistically significant, the correlation coefficient between OBCFA and *S. ruminantium, R. amylophilus*, and *S. bovis* populations were all lower than.50.

## Discussion

The concentration of *anteiso*-C15:0 (17.6–23.6%) and C15:0 (15.4–38.1%) observed in the current study are consistent with those (*anteiso*-C15:0, 24.7–29.6%; C15:0, 24.9–27.9%) observed by the study of Vlaeminck et al. (1) in fermented mixtures after 21 h incubation (*in vitro*) of four types of dairy diets. Moreover, the concentration of *anteiso*-C15:0 and C15:0 observed in this study was consistent with those observed in rumen bacteria (*anteiso*-C15:0, 23.76%; C15:0, 25.7%) collected from Holstein cows under different feeding regimes (24, 25). The study of Vlaeminck et al. (26) reported a close relationship (*r* = −0.77) between *anteiso*-C15:0 concentration and dietary forage inclusion levels in the diet of dairy cows. However, large changes in *anteiso*-C15:0 concentrations were not observed in this study with increasing levels of cellulose during *in vitro* incubation. Generally, the odd-chain fatty acids were formed using propionate as a primer (4). As for the branched-chain fatty acids, the *iso*-fatty acids were produced from isobutyrate and isovalerate, while *anteiso*-fatty acids originated from 2-methybutyrate in ruminants (12). The synthesis of these short-chain fatty acids was not only influenced by diet composition and feeding strategies (3, 27), but also highly dependent on the ruminal environment and physiological conditions of dairy cows (28). Indeed, studies regarding ruminal OBCFA synthesis in cows fed rations with different ratios of forage and concentrate have shown contrasting results. With increasing inclusion levels of forages in the diet, total OBCFA synthesis has been reported to be linearly increased in the study of Vlaeminck et al. (26). However, the study of Zhang et al. (3) observed that with increasing inclusion levels of forages in the diet OBCFA synthesis changed quadratically in rumen contents after 6 h morning feeding, which is partially in line with our data. As known, the OBCFA, especially BCFA, was mainly derived from bacteria and they are the unique components of cell membrane lipids in bacteria (4), but also exist in rumen protozoa (24, 25). Therefore, the increase in OBCFA synthesis during the 24 h incubation in the present study, indicated that the microorganisms were keeping a strong reproducibility and proliferation during the whole *in vitro* incubation. This represented a good condition for microbial metabolic process and fermentation activities. However, the decreases in ruminal OBCFA synthesis during 12–17 h after feeding (2, 29) mainly disagree with our results. This might be due to the characteristics of the ruminal *in vitro* culture system, in which the fermentation products, e.g., VFAs, could not outflow nor be absorbed, as done by rumen mucosal epithelia, which might have contributed to the accumulation of OBCFA over time, as observed in our study.

The observed VFA profiles in the present study are in accordance with recent studies of Wang et al. (23) and Li et al. (30) who all reported that increasing dietary forage proportion decreased TVFA production in the rumen of dairy cows. The increasing proportion of fermented carbohydrates, such as starch, enhanced the microbial population and they capture more of the released NH_3_-N to synthesize MCP (30), which partly explained our results. As well-known, the short-chain fatty acids (e.g., VFA) produced during rumen fermentation were readily absorbed by rumen epithelial papilla in ruminants (31). However, failure of VFA absorption in the batch *in vitro* system resulted in higher accumulation of VFA and a consequent decrease in pH values as incubation time progresses, which explained our findings. Furthermore, the decrease in NH_3_-N concentrations overtime might suggest that these released nutrients were utilized by microorganisms, which corresponded well with the increased production of MCP overtime during the 24 h of incubation in our *in vitro* study.

Increasing dietary forage proportion promotes the growth of cellulose-degrading bacteria and reduces the relative abundance of amylolytic bacteria in the rumen (23, 32), which supported the findings of our study. The observed reductions of the relative abundance of all the bacteria (except *S. ruminantium*) during 24 h incubation indicated microbial lysis and decomposition, as previously reported for a batch *in vitro* system by the study of Wells and Russell (33). Moreover, the study of Cone et al. (34) observed that microbial activities in rumen fluid stored anaerobically at 39°C for 24 h was considerably decreased as compared with fresh inoculum.

Rumen fermentation products, namely short-chain fatty acids, are usually regarded as precursors for estimation of OBCFA synthesis (12). The quantities of OBCFA have been reported to be a potential predictor for microbial growth (2), since these fatty acids, especially branched-chain fatty acids, are the consistent components of bacterial membrane lipids (4). However, studies exploring the relationship between rumen VFA and OBCFA, have shown mixed results. Despite this, our results are in part consistent with previous findings of an *in vitro* incubation trial with nine mixed dairy cow rations (1), which reported a negative correlation of acetate with C15:0 and *iso*-C17:0, and a positive correlation of propionate with C15:0. The study of French et al. (6) observed that ruminal OBCFA response was minor, following infusion of large amounts of acetate and propionate. The findings of this study extrapolated that extracellular VFA concentrations did not alter rumen OBCFA concentration. Furthermore, the rumen OBCFA profiles have been reported to be largely determined by the relative abundance of specific microbial strains rather than the availability of precursors (12, 35).

The correlations of OBCFA concentrations with NH_3_-N should be largely interpreted concerning the utilization and incorporation of NH_3_-N by rumen microorganisms into MCP, which contain OBCFA in their cell membranes (4). Similarly, the study of Zhang et al. (3) showed that there was a negative relationship between NH_3_-N and OBCFA in the rumen contents of Holstein cows. Even though data on MCP synthesis was not available in previous works of literature, indirect comparisons could be made. For example, the studies conducted by Vlaeminck et al. (2) and Liu et al. (8) demonstrated that the total OBCFA or the sum of some specific OBCFA, e.g., C13:0, C15:0, *iso*-C15:0, and C17:0, was closely related to microbial markers, such as uracil and purine bases (2), and pyrimidine (8). The synthesis of microbial protein is a very complex biological process, which is related to many factors such as the activity of the different enzymes, VFA production and ammonia utilization. All these factors influence the activities of the microorganisms, including their reproduction and lysis as well as OBCFA concentration which are present in microbial membrane lipids. Therefore, the strong correlation of MCP concentration with iso-C16:0 and *anteiso*-C17:0 concentrations observed in our study indicated that these two fatty acids, especially *iso*-C16:0 were a good index for estimation of microbial flow from the rumen.

Changes in rumen microbial population, which were driven by altered diet composition or environmental conditions (36, 37), might lead to variations in OBCFA concentrations. Based on the available data from pure culture studies, it was evident that the OBCFA compositions were always species-specific (10, 38, 39), or even varied widely in microbial strains (11, 39). Some strains of *R. albus* spp., e.g., *R albus* 7, contained a high amount of linear odd-chain fatty acids (40), and this is also supported by the positive correlation between the concentration of C15:0 and TOCFA with *R. albus* relative abundance in the present study. Several studies have reported that *iso*-C15:0 is the predominant OBCFA in the pure cultured strains of *R. flavefaciens* (*R. flavefaciens* FD1 and C94), accounting for 37–41% of total fatty acids (35, 40). Combining these results, along with the positive correlation between *iso*-C15:0 and *R. flavefaciens* in our experiment, indicated that more abundance of this bacteria might produce a high level of *iso*-acids in the rumen. Increasing dietary forage provision would lead to the rapid growth of cellulolytic bacteria such as *R. flavefaciens* (23, 41), and results in higher production of *iso*-C15:0 in the rumen, as expected (3). However, the strongest correlation of *R. flavefaciens* was with C15:0 concentration in the present study, which seemed to be discrepant with measured cellular fatty acid compositions in pure culture studies (35, 40). As summarized by the study of Vlaeminck et al. (12), *B. fibrisolvens* had a heterogeneous profile of OBCFA, which might partly explain the negative relationship observed in our study. The observed weak to moderate correlations between amylolytic bacteria and OBCFA in the present study are consistent with the results of the study by Zhang et al. (3), who found a negative correlation between *S. ruminantium* with *anteiso*-C17:0 (ρ = 0.21, *P* < 0.05), and a positive relationship between *S. bovis* and C15:0 (ρ = 0.44, *P* < 0.05) and total OBCFA (ρ = 0.37, *P* < 0.05) in 108 rumen samples obtained from dairy cows fed rations with different ratios of forage and concentrate.

## Conclusions

*In vitro* ruminal incubation of pure carbohydrate substrates with different C:S ratios, i.e., 0:100, 25:75, 50:50, 75:25, and 100:0, produced different amounts of OBCFA. Except for *iso*-C17:0, the concentrations of all individual OBCFA were affected (*P* < 0.05) by the interaction of the C:S ratio and incubation time. The highest concentration of total OBCFA (3.94 mg/g DM) was observed in the fermented mixture after 24 h incubation when the C:S was 0:100, while the lowest concentration of OBCFA (1.65 mg/g DM) was produced after 6 h of incubation when the C:S was 50:50. The odd- and branched-chain fatty acids concentrations were closely correlated to rumen VFA, MCP, and bacterial populations. In particular, the *iso*-C16:0 concentration might have potential as a marker of estimation of total VFA and MCP concentrations with ρ being.78 and.82, respectively. Compared with starch degrading bacteria, cellulolytic bacteria had relatively stronger correlations with OBCFA profiles. However, to achieve a final accurate conclusion on the relationship of OBCFA with rumen fermentation profiles, more in-depth experiments are needed to substantiate the current findings.

## Data Availability Statement

The original contributions presented in the study are included in the article/supplementary material, further inquiries can be directed to the corresponding authors.

## Ethics Statement

The animal study was reviewed and approved by the Ethical Committee of the College of Animal Science and Technology of Northeast Agricultural University.

## Author Contributions

HX, NK, XLiu, and XLi: supervision, conceptualization, funding acquisition, project administration, and writing of the original draft. XJ, FS, SZ, YS, and YZ: conceived, designed investigation, conceptualization and formal analysis, review, and editing. All authors contributed to the article and approved the submitted version.

## Funding

This study was supported by the National Natural Science Foundation of China (Grant No. 31702135) and the China Agricultural Research System of MOF and MARA and Heilongjiang Provincial Dairy Industry and Technology System.

## Conflict of Interest

XLiu and SZ were employed by the company Wellhope Feed Company Limited. The remaining authors declare that the research was conducted in the absence of any commercial or financial relationships that could be construed as a potential conflict of interest.

## Publisher's Note

All claims expressed in this article are solely those of the authors and do not necessarily represent those of their affiliated organizations, or those of the publisher, the editors and the reviewers. Any product that may be evaluated in this article, or claim that may be made by its manufacturer, is not guaranteed or endorsed by the publisher.

## Abbreviations

*anteiso*-C15:0: 12-methyltetradecanoic acid
*anteiso*-C17:0: 14-methylhexadecanoic acid
C15:0: pentadecanoic acid
C17:0: heptadecanoic acid
DM: dry matter
C:S: cellulose:starch ratio
*iso*-C15:0: 13-methyltetradecanoic acid
*iso*-C16:0: 14-methylpentadecanoic acid
*iso*-C17:0: 15-methylhexadecanoic acid
MCP: microbial crude protein
NH_3_-N: ammonia nitrogen
OBCFA: odd- and branched-chain fatty acids
TCA: trichloroacetic acid
TIFA: total *iso*-fatty acids
TAFA: total *anteiso*-fatty acids
TOCFA: total odd-chain fatty acids
TBCFA: total branched-chain fatty acids
TVFA: total volatile fatty acids
VFA: volatile fatty acids.
